# Supplementary material for: Unidirectional MCM translocation away from ORC drives origin licensing
Source: Nat Commun. 2025 Jan 17;16:782. doi: 10.1038/s41467-025-56143-y (PMC11748629; doi:10.1038/s41467-025-56143-y)
Supplement: Supplementary file 1 — Supplementary Information [file 41467_2025_56143_MOESM1_ESM.pdf]

Supplementary Information for

Unidirectional MCM translocation away from ORC drives origin licensing

Agata Butryn[1], Julia F. Greiwe[1,2], Alessandro Costa[1,*]

[1] Macromolecular Machines Laboratory, The Francis Crick Institute, London NW1 1AT, U.K.

[2] Current address: Astex Pharmaceuticals,436 Cambridge Science Park Milton Rd, Milton, Cambridge CB4 0QA, U.K.

* Correspondence to A.C. alessandro.costa@crick.ac.uk

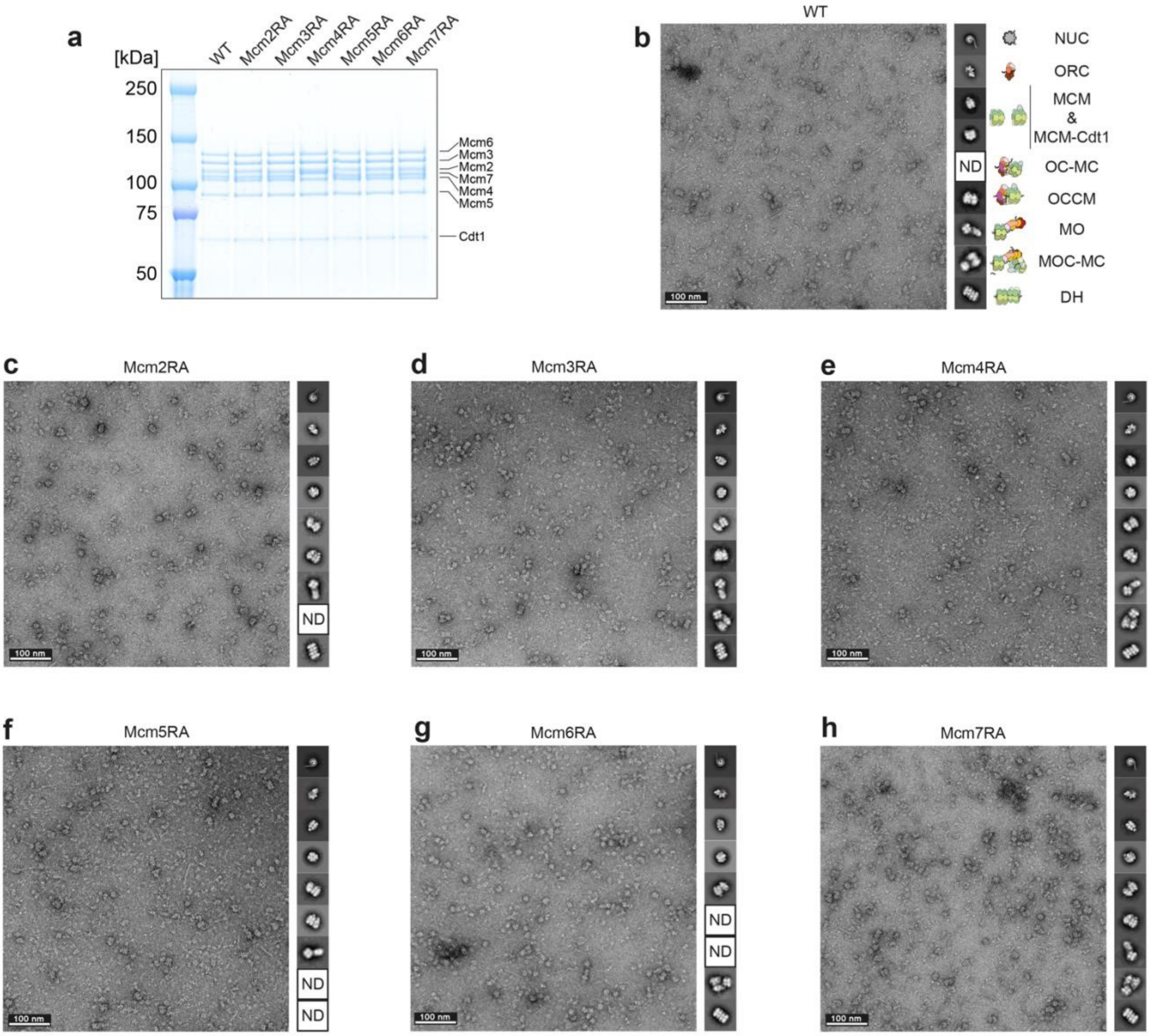

Supplementary Figure 1. Impact of Arginine Finger mutations on MCM-Cdt1 loading on origin DNA in solution. a Coomassie-stained SDS-PAGE analysis of purified MCM-Cdt1 complexes from RA mutant proteins used in this study**.** Source data are provided as a Source Data file. This experiment was performed once. **b-h** Representative negative stain micrographs and 2D class averages of DH loading reactions performed with wild-type (WT) MCM-Cdt1 and MCM RA mutant proteins. NUC: nucleosome. OC-MC: ORC-Cdc6-MCM-Cdt1 complex. OCCM: ORC-Cdc6-Cdt1MCM complex. MO: MCM-ORC complex. MOC-MC: MCM-ORC-Cdc6-MCM-Cdt1 complex. DH: MCM double hexamer. ND: not detected. Approximately 100 micrographs were collected for each dataset.

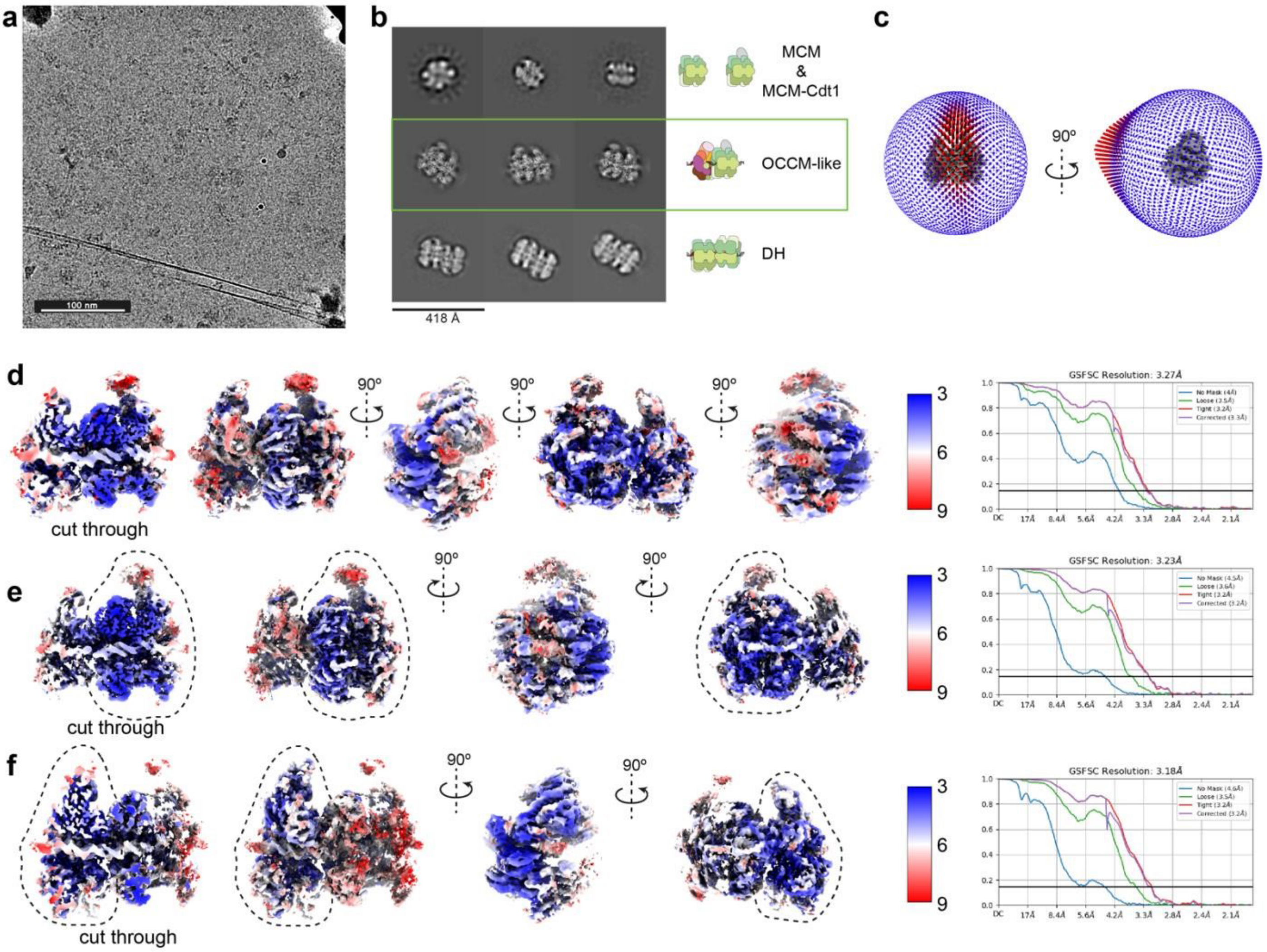

Supplementary Figure 2. Cryo-EM analysis of Mcm2RA mutant complexes. Representative micrograph (**a**) and 2D class averages (**b**) of observed protein assemblies. 63,858 micrographs were collected in total. **c** 3D representation of angular particle distribution within $OCCM^{Mcm2RA}$ particles, calculated using cryoSPARC[1]. **d-f** final cryo-EM density maps color-coded by local resolution and the gold-standard Fourier shell correlation (GSFSC) plots computed in cryoSPARC[1] for: global refinement of $OCCM^{Mcm2RA}$ (**d**), local refinement of MCM-Cdt1 ring (**e**) and local refinement of ORC complex (**f**). Dashed lines in **e** and **f** indicate the masks used for local refinements.

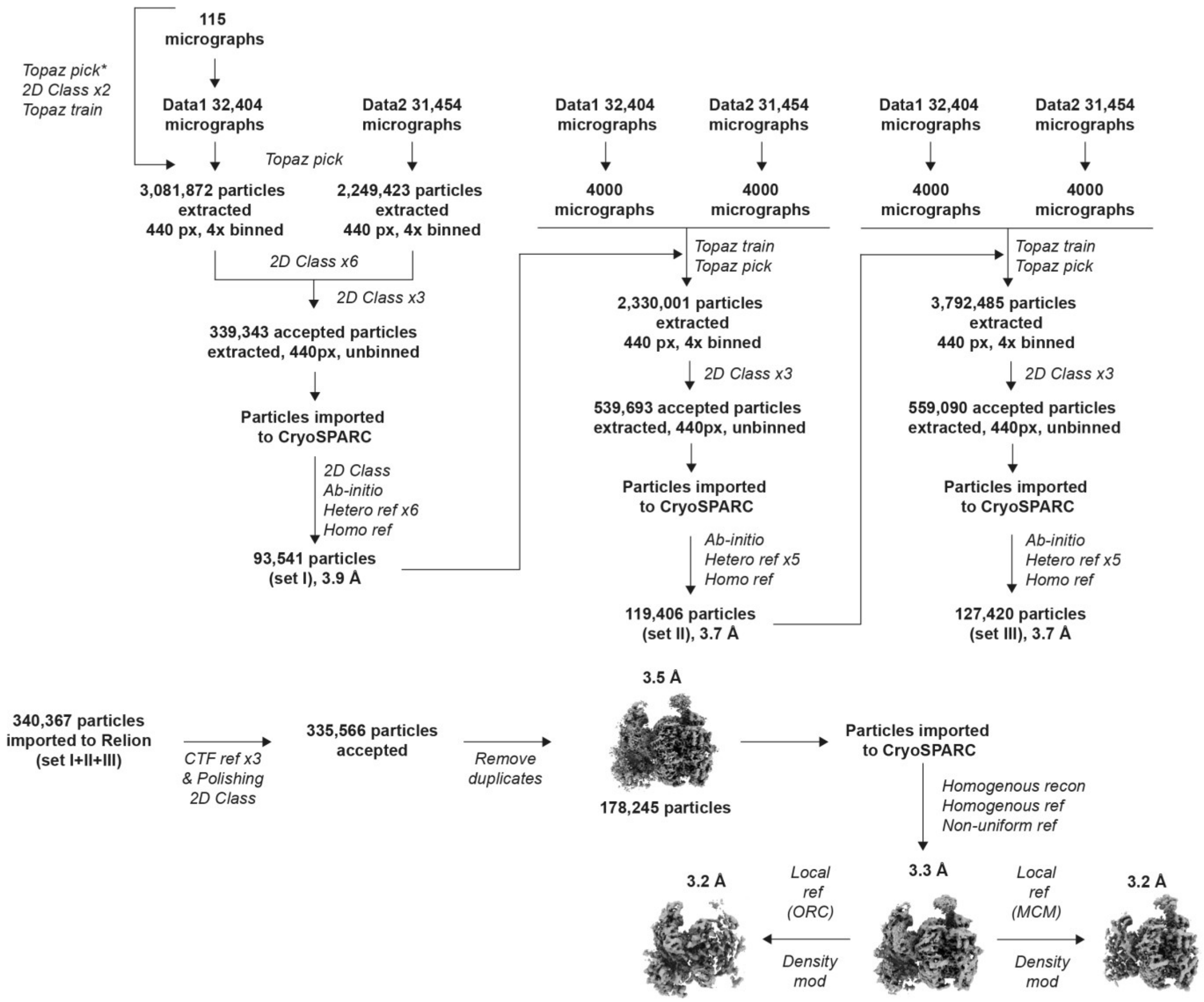

Supplementary Figure 3. Image processing. Flowchart outlining the cryo-EM image processing pipeline for OCCM^{Mcm2RA} complexes.

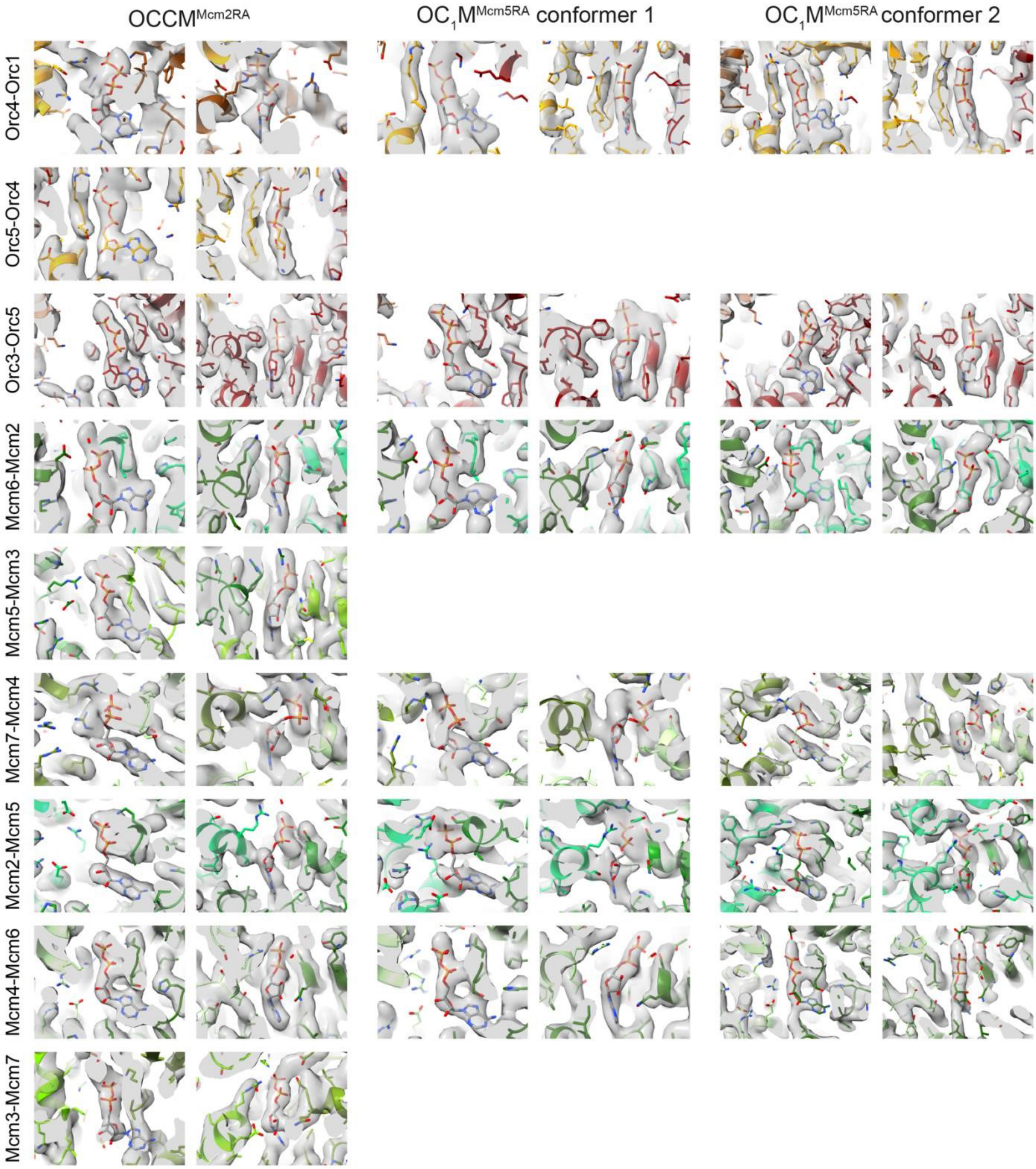

Supplementary Figure 4. Cryo-EM density of nucleotides and their residue environment in the different ATPase sites in all structures generated in this study. Two views of each occupied ATPase site are shown.

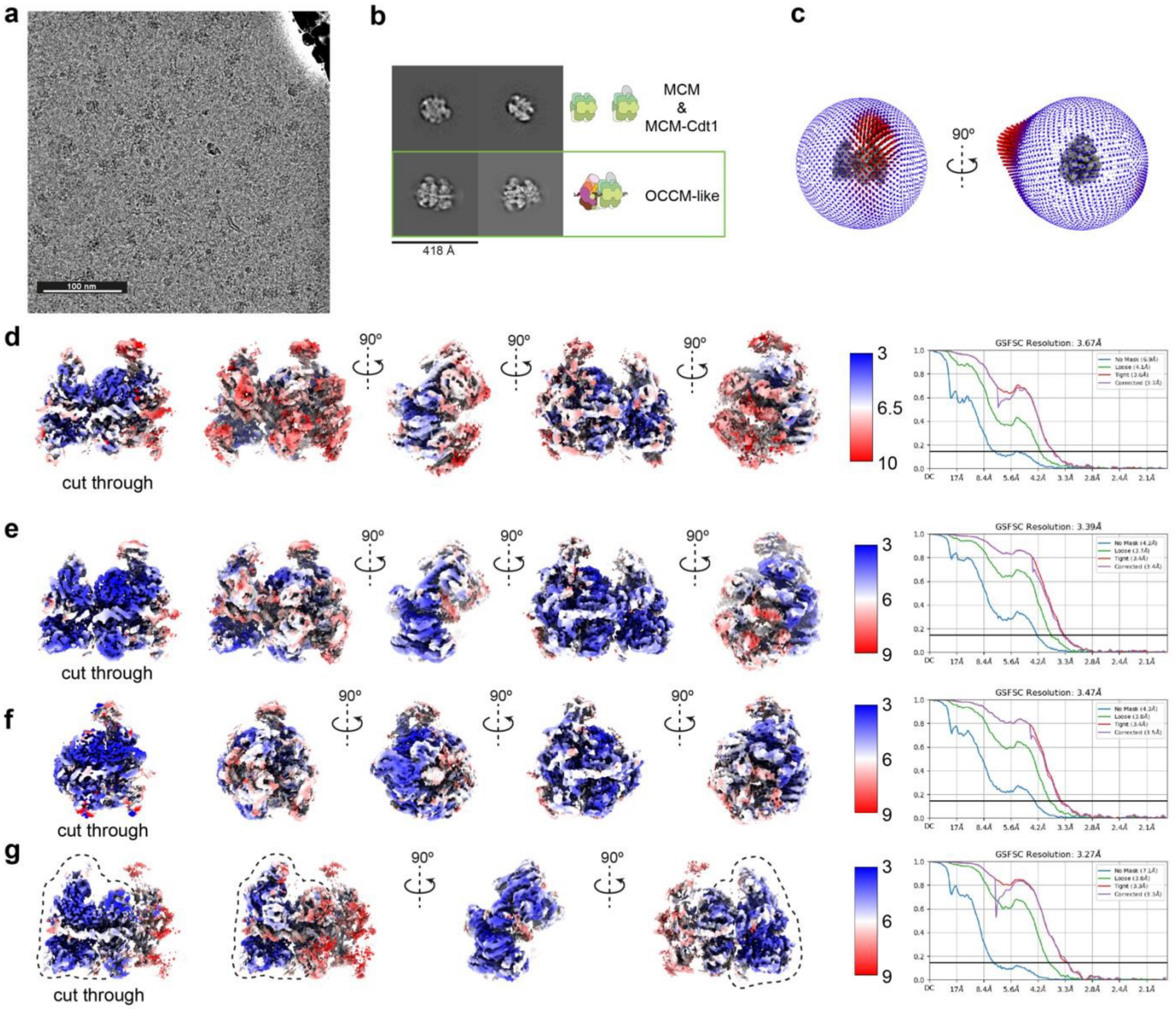

Supplementary Figure 5. Cryo-EM analysis of Mcm5RA mutant complexes. Representative micrograph (**a**) and 2D class averages (**b**) of observed protein assemblies. 29,191 micrographs were collected in total. **c** 3D representation of angular particle distribution within OC_1M^{Mcm5RA} particles, calculated using cryoSPARC[1] **d-g.** Final cryo-EM density maps color-coded by local resolution and the gold-standard Fourier shell correlation (GSFSC) plots computed in cryoSPARC[1] for the final maps: global refinement of OC_1M^{Mcm5RA} conformer 1 (**d**), global refinement of OC_1M^{Mcm5RA} conformer 2 (**e**) refinement of MCM-Cdt1 ring in conformer 2 after particle subtraction (**f**) and local refinement of ORC complex in conformer 2 (**g**). Dashed lines in **g** indicate the mask used for local refinement of the ORC complex.

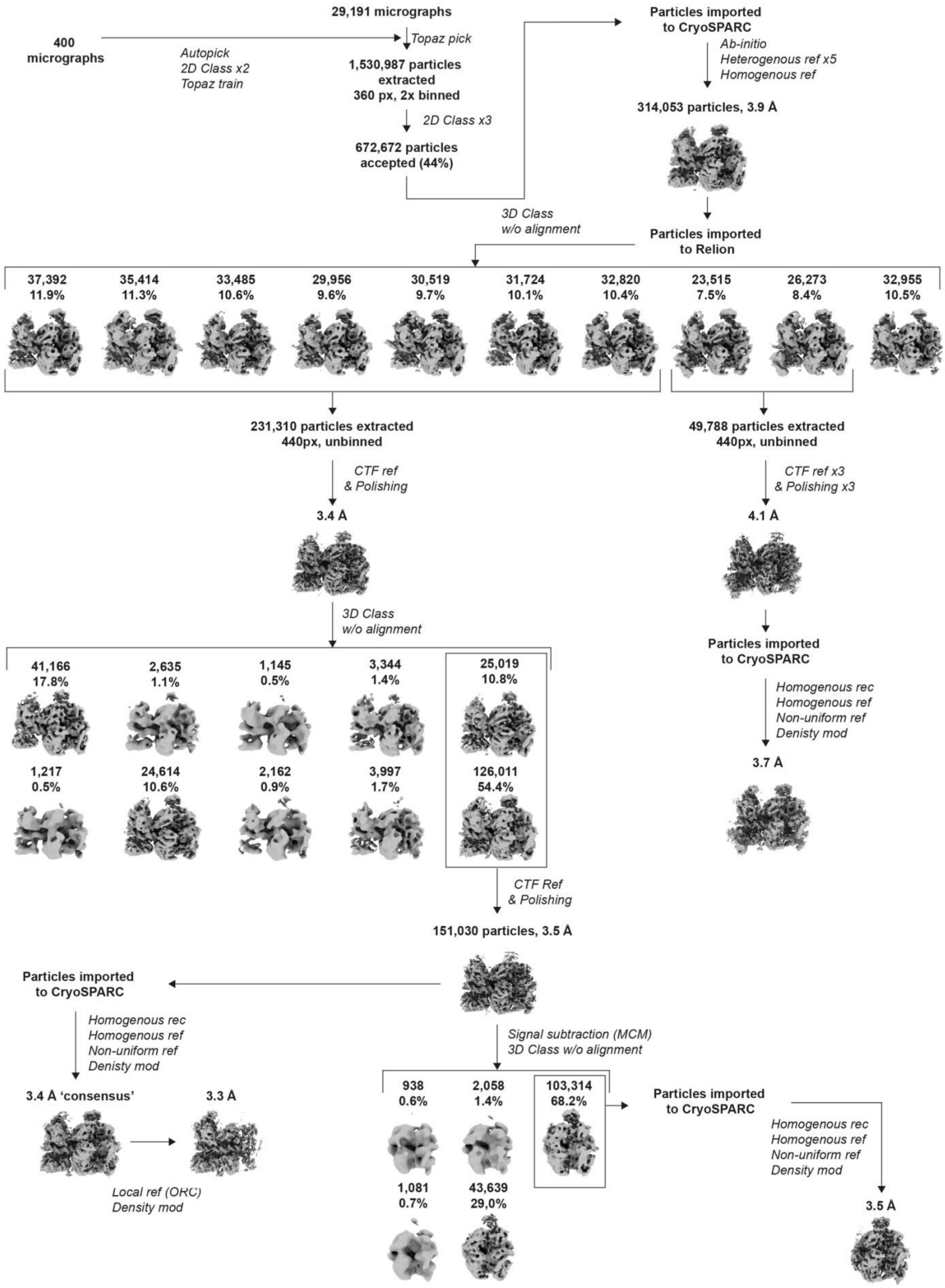

29,191 micrographs
400 micrographs
Autopick
2D Class x2
Topaz train
Topaz pick
1,530,987 particles extracted
360 px, 2x binned
2D Class x3
672,672 particles accepted (44%)
Particles imported to CryoSPARC
Ab-initio
Heterogenous ref x5
Homogenous ref
314,053 particles, 3.9 Å
3D Class w/o alignment
Particles imported to Relion
37,392 11.9%
35,414 11.3%
33,485 10.6%
29,956 9.6%
30,519 9.7%
31,724 10.1%
32,820 10.4%
23,515 7.5%
26,273 8.4%
32,955 10.5%
231,310 particles extracted
440px, unbinned
CTF ref
& Polishing
3.4 Å
3D Class
w/o alignment
41,166 17.8%
2,635 1.1%
1,145 0.5%
3,344 1.4%
25,019 10.8%
1,217 0.5%
24,614 10.6%
2,162 0.9%
3,997 1.7%
126,011 54.4%
49,788 particles extracted
440px, unbinned
CTF ref x3
& Polishing x3
4.1 Å
Particles imported to CryoSPARC
Homogenous rec
Homogenous ref
Non-uniform ref
Denisty mod
3.7 Å
CTF Ref
& Polishing
151,030 particles, 3.5 Å
Particles imported to CryoSPARC
Homogenous rec
Homogenous ref
Non-uniform ref
Denisty mod
3.4 Å 'consensus'
3.3 Å
Local ref (ORC)
Density mod
Signal subtraction (MCM)
3D Class w/o alignment
938 0.6%
2,058 1.4%
103,314 68.2%
1,081 0.7%
43,639 29,0%
Particles imported to CryoSPARC
Homogenous rec
Homogenous ref
Non-uniform ref
Density mod
3.5 Å

Supplementary Figure 6. Image processing. Flowchart outlining the cryo-EM data processing pipeline for OC_1M^{Mcm5RA} complexes.

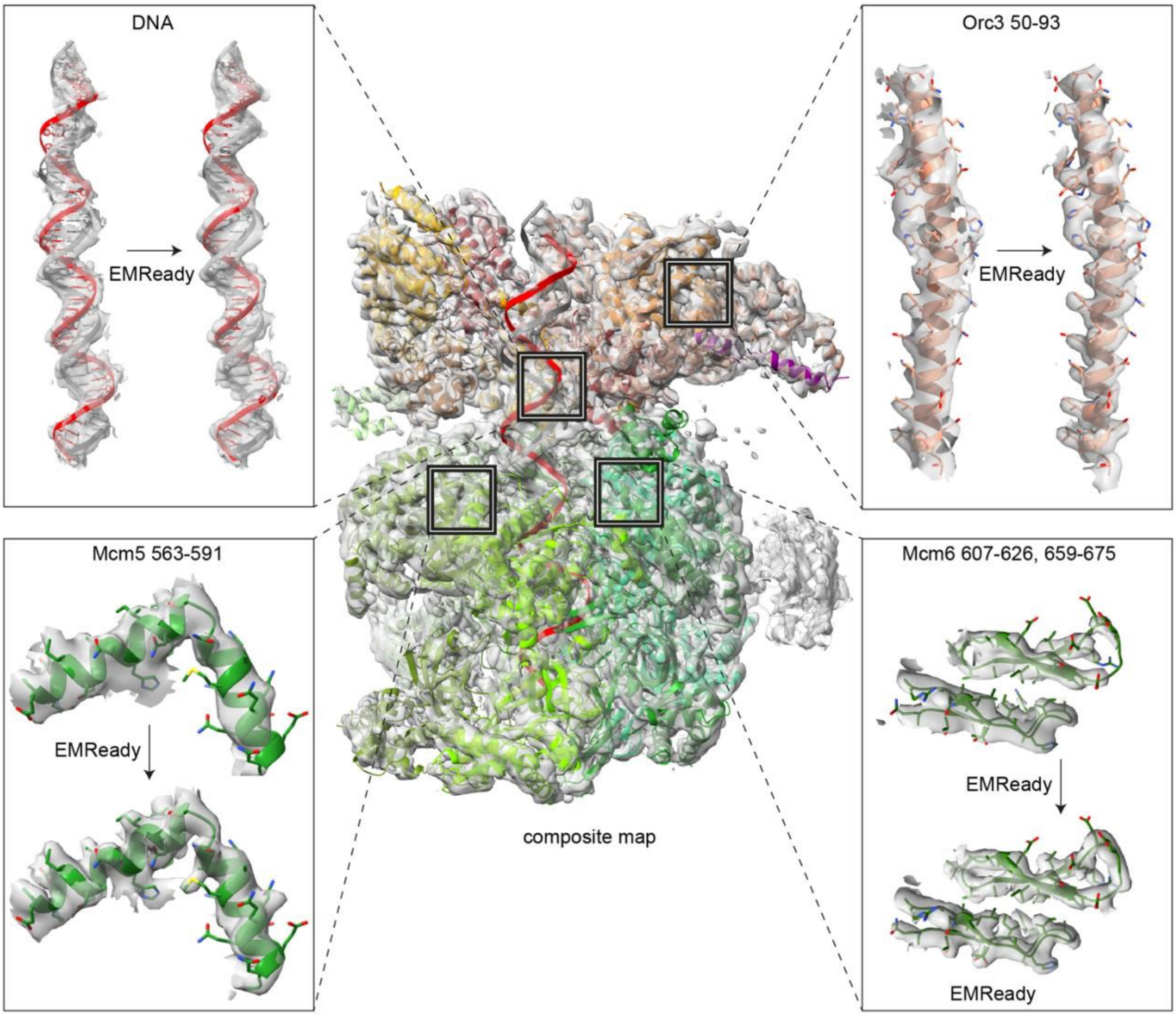

Supplementary Figure 7. Representative regions of the cryo-EM map of the OC_1M^{Mcm5RA} conformer 2. The central panel displays a composite map (transparent surface, zoned 3.5 Å and 2.5 Å within DNA and protein atoms, respectively) created by combining locally refined unmodified maps of the ORC and MCM-Cdt1 subcomplexes (see Materials for details). Side panels show the quality of the composite map and map after applying density modification with EMReady[2].

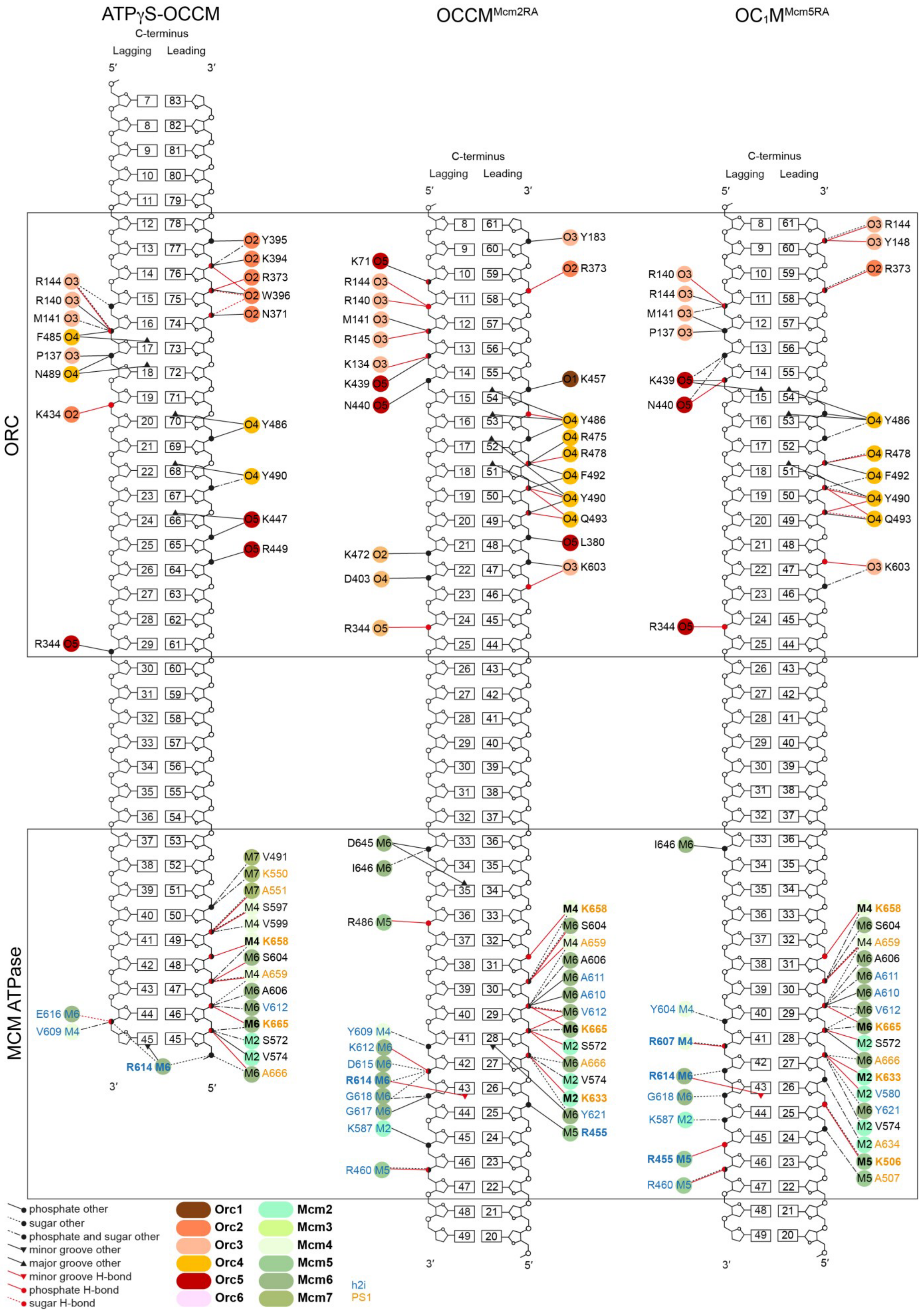

ATPγS-OCCM
OCCM^Mcm2RA
OC1M^Mcm5RA
C-terminus
Lagging
Leading
5′
3′
ORC
MCM ATPase
O2 Y395
O2 K394
O2 R373
O2 W396
O2 N371
R144 O3
R140 O3
M141 O3
F485 O4
P137 O3
N489 O4
K434 O2
O4 Y486
O4 Y490
O5 K447
O5 R449
R344 O5
M7 V491
M7 K550
M7 A551
M4 S597
M4 V599
M4 K658
M6 S604
M4 A659
M6 A606
M6 V612
M6 K665
M2 S572
M2 V574
M6 A666
E616 M6
V609 M4
R614 M6
O3 Y183
O2 R373
K71 O5
R144 O3
R140 O3
M141 O3
R145 O3
K134 O3
K439 O5
N440 O5
O1 K457
O4 Y486
O4 R475
O4 R478
O4 F492
O4 Y490
O4 Q493
O5 L380
O3 K603
K472 O2
D403 O4
R344 O5
D645 M6
I646 M6
R486 M5
M4 K658
M6 S604
M4 A659
M6 A606
M6 A611
M6 A610
M6 V612
M6 K665
M2 S572
M6 A666
M2 V574
M2 K633
M6 Y621
M5 R455
Y609 M4
K612 M6
D615 M6
R614 M6
G618 M6
G617 M6
K587 M2
R460 M5
O3 R144
O3 Y148
O2 R373
R140 O3
R144 O3
M141 O3
P137 O3
K439 O5
N440 O5
O4 Y486
O4 R478
O4 F492
O4 Y490
O4 Q493
O3 K603
R344 O5
I646 M6
M4 K658
M6 S604
M4 A659
M6 A606
M6 A611
M6 A610
M6 V612
M6 K665
M2 S572
M6 A666
M2 K633
M2 V580
M6 Y621
M2 V574
M2 A634
M5 K506
M5 A507
Y604 M4
R607 M4
R614 M6
G618 M6
K587 M2
R455 M5
R460 M5
phosphate other
sugar other
phosphate and sugar other
minor groove other
major groove other
minor groove H-bond
phosphate H-bond
sugar H-bond
Orc1
Orc2
Orc3
Orc4
Orc5
Orc6
Mcm2
Mcm3
Mcm4
Mcm5
Mcm6
Mcm7
h2i
PS1

Supplementary Figure 8. Schematic representation of DNA-protein interactions in ATPγSOCCM (PDB entry 5V8F[3]), OCCM^{Mcm2RA} (PDB 9GJW), and conformer 2 OC$_1$M^{Mcm5RA} (PDB 9GJP). DNA-protein interactions were calculated using DNAproDB[4] and for clarity visualized showing only contacts within 4 Å. Structures are aligned by base position for easier comparison. Protein components and interaction types are defined in the legend. Protein residues that were characterized biochemically in this study are highlighted in bold. H2i: h2i hairpin. PS1: PS1 hairpin.

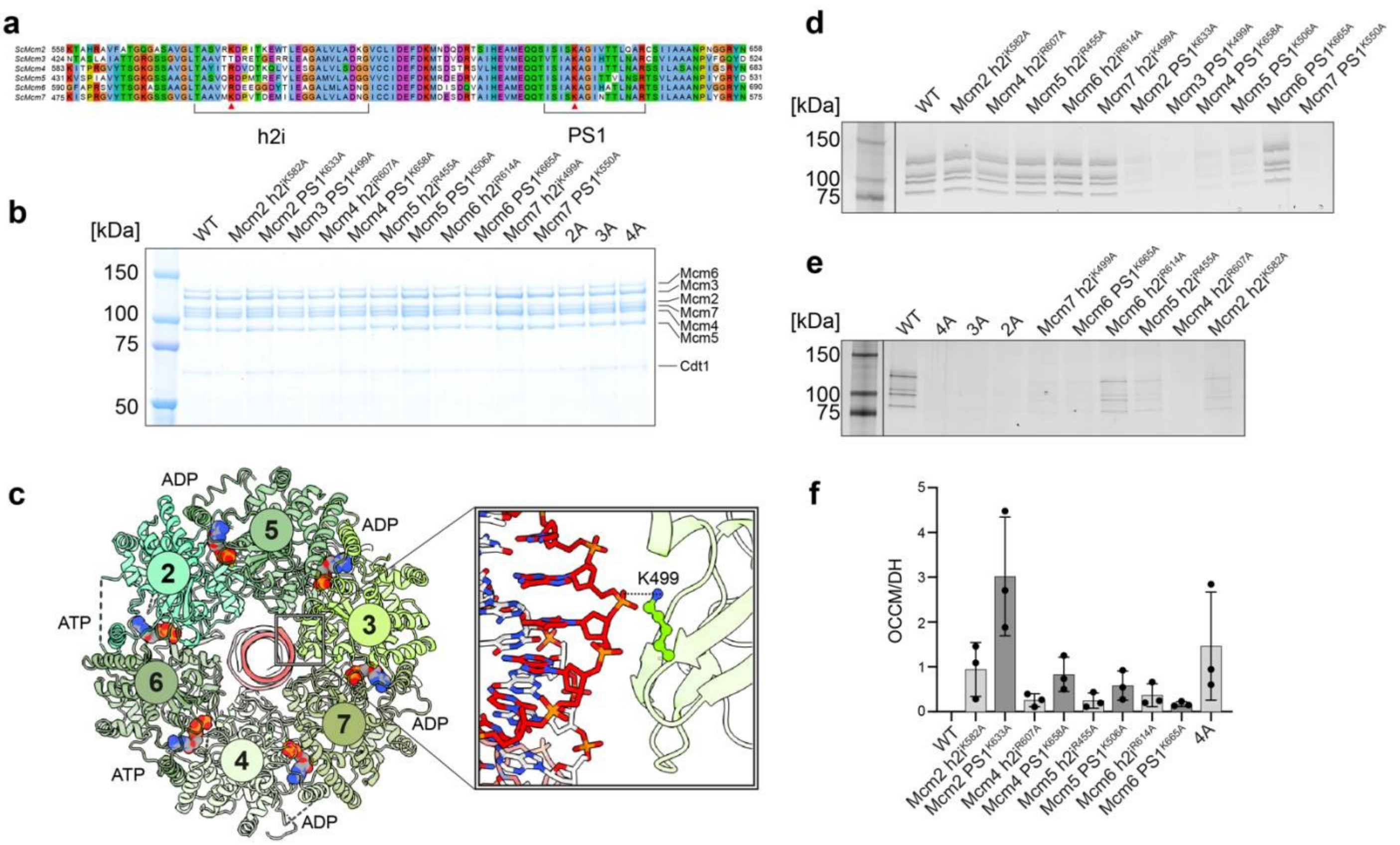

Supplementary Figure 9. Characterization of MCM-Cdt1 complexes and DH formation from MCM H2i and PS1 hairpin mutants. a Sequence alignment of *Saccharomyces cerevisiae* Mcm hairpins. **b** Coomassie-stained SDS-PAGE analysis of purified MCM-Cdt1 complexes from MCM h2i and PS1 hairpin mutant proteins used in this study. **c** Structure of a single-loaded MCM hexamer (PDB entry 8RIG[5]). K499 of the Mcm3 PS1 hairpin engages in a hydrogen bond with a backbone phosphate of the leading-strand template. **d** Silver-stained SDS-PAGE analysis of DH complexes pulled down using streptavidin-coated magnetic beads and a roadblocked ARS1 origin template, formed with MCM h2i and PS1 hairpin mutants, performed with higher ATP (5 mM) and MCM-Cdt1 (550 nM) concentrations. This experiment was performed once. **e** Silver-stained SDS-PAGE analysis as in **d** but performed at lower ATP (315 µM) and MCM-Cdt1 (220 nM) concentrations, showing selected MCM h2i and PS1BH mutants. 2A: $Mcm2^{K582A}$-$Mcm5^{R455A}$, 3A: $Mcm2^{K582A}$$Mcm5_{R455A}$-$Mcm6_{R614A}$, 4A: $Mcm2_{K582A}$-$Mcm4_{R607A}$-$Mcm5_{R455A}$-$Mcm6_{R614A}$. This experiment was performed once. **f** Analysis of DH loading reactions shown in **Figure 6c** displayed as OCCM/DH ratio, with bar graphs showing mean ± SD. N=3 independent experiments. Source data for panels **b**, **d**, **e**, and **f** are provided as a Source Data file.

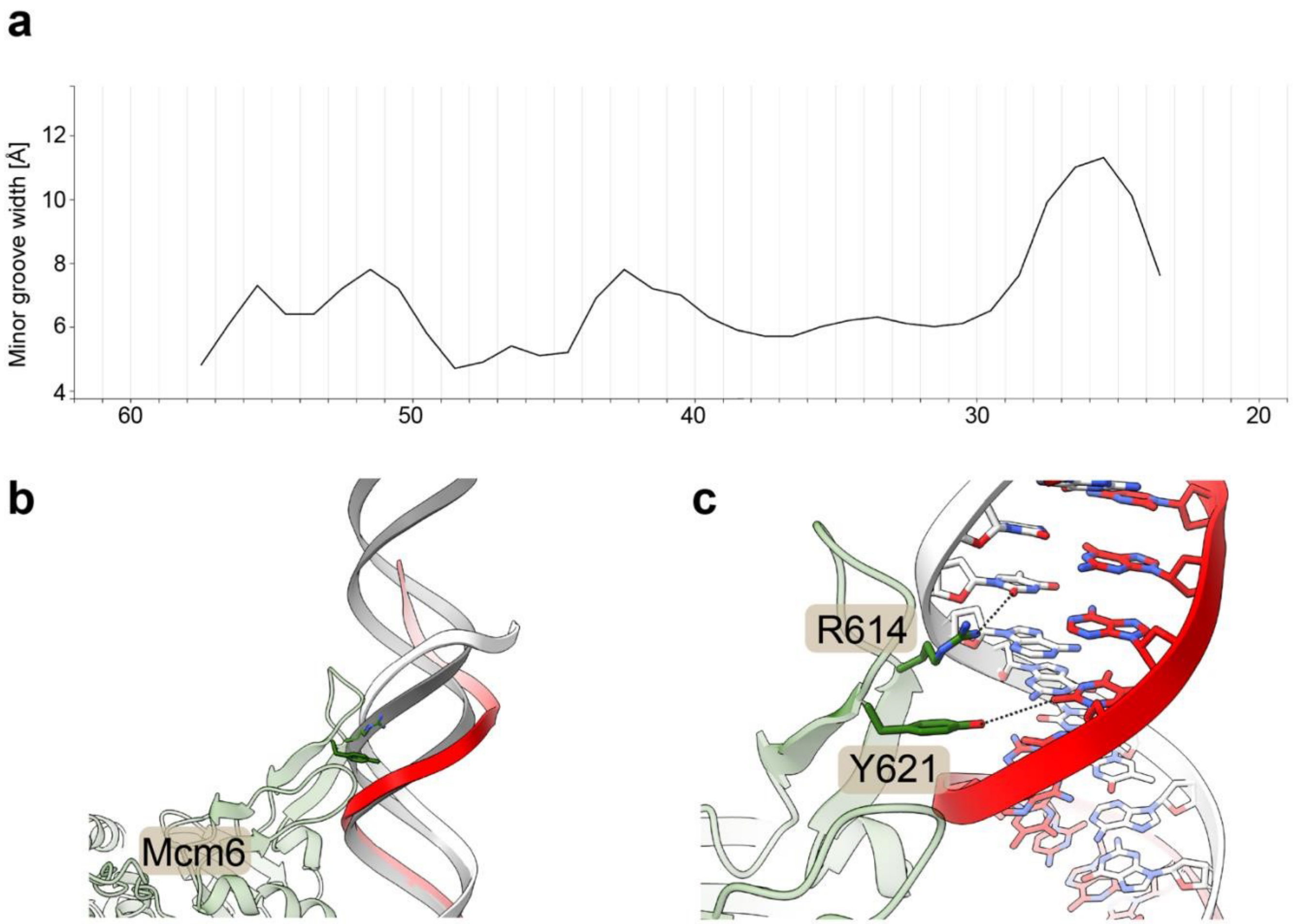

Supplementary Figure 10. Minor groove widening as observed in OC$_1$M^{Mcm5RA} complex. a Minor groove width in conformer 2 OC$_1$M^{Mcm5RA} structure computed by the DNAproDB server[4]. X axis labels correspond to the leading-strand template residue numbering. **b-c** Close-up view on Mcm6 pore loops. **b** H2i insertion penetrates minor groove causing a slight kink in downstream DNA, as compared to ideal B-form DNA (grey). **c** Mcm6 h2i insertion residues R614 and Y621 engage in hydrogen bonding with DNA bases.

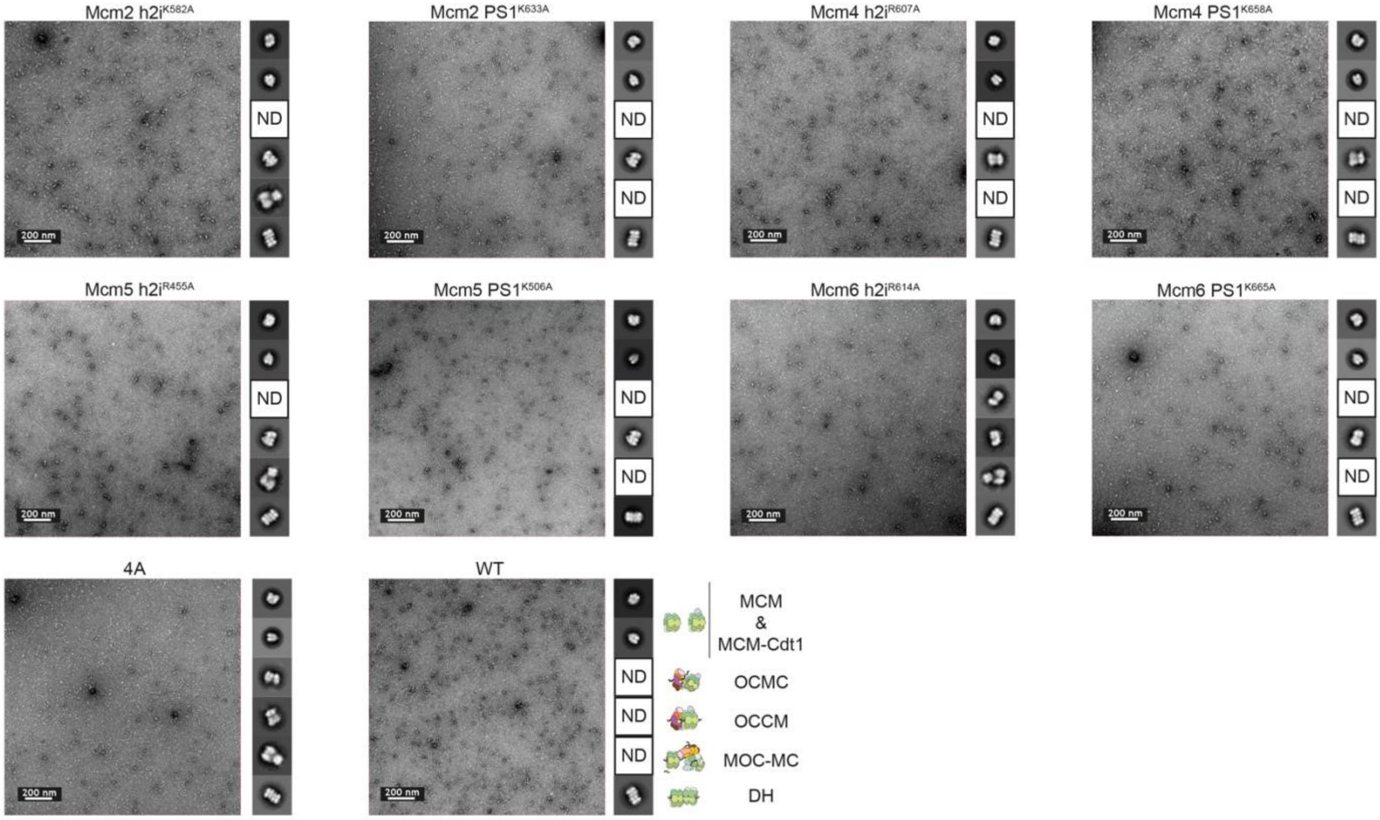

Supplementary Figure 11. Impact of h2i and PS1 hairpin mutations on MCM-Cdt1 Loading on origin DNA in solution. Representative negative stain micrographs and class averages are shown. OC-MC: ORC-Cdc6-MCM-Cdt1 complex. OCCM: ORC-Cdc6-Cdt1-MCM complex. MOC-MC: MCM-ORC-Cdc6-MCM-Cdt1 complex. DH: MCM double hexamer. ND: not detected. Approximately 50 micrographs were collected for each dataset.

Supplementary Table 1. Data collection and refinement statistics for the Mcm2RA dataset.

	ORC local refinement EMDB-51405	MCM-Cdt1 local refinement EMDB-51406	OCCM^{Mcm2RA} global refinement EMD-51404 (consensus), EMD-51407 (composite) PDB 9GJW
**Data collection and processing**			
Magnification	130,000	130,000	130,000
Voltage (kV)	300	300	300
Electron exposure (e^{-}/Å^2)	30.34 (data1), 30.0 (data2)	30.34 (data1), 30.0 (data2)	30.34 (data1), 30.0 (data2)
Defocus range (μm)	-1.5 to -2.7	-1.5 to -2.7	-1.5 to -2.7
Pixel size (Å)	0.95	0.95	0.95
Final particle images (no.)	178,245	178,245	178,245
Map resolution (Å) at 0.143 FSC threshold	3.18	3.23	3.27
**Refinement**			
Model-map CC[a,b] (CC_{mask}/CC_{box}/CC_{peaks}/CC_{volume})			0.65/0.77/0.52/0.65
Model resolution (Å) at 0.143 FSC threshold[a,b] (masked/unmasked)			3.3/3.4
Model resolution range (Å)[c]			2.9-11.0
Model composition			
Non-hydrogen atoms			52,380
Protein residues			6,291
Ligands			3 ATP, 6 ADP, 4 Zn^{2+}
B factors (Å^2)[a]			
Protein			245.95
Ligand			199.31
R.m.s. deviations[a]			
Bond lengths (Å)			0.002
Bond angles (°)			0.539
Validation[d]			
MolProbity score			1.19
Clashscore			4.00
Poor rotamers (%)			0.04
CaBLAM outliers (%)			0.96
Ramachandran plot[d]			
Favored (%)			98.05
Allowed (%)			1.95
Disallowed (%)			0

[a] Comprehensive validation (cryo-EM) in Phenix[6]. [b] Values shown are for the consensus map, which was used for the refinement. [c] Estimated with Chimera 'Values at atom positions' tool[7].
[d] MolProbity[8] validation in Phenix.

Supplementary Table 2. Data collection and refinement statistics for the Mcm5RA dataset.

	OC_1M_{Mcm5RA} conformer 1 global refinement EMD-51441 (consensus) PDB 9GM5	Conformer 2 MCM-Cdt1 local refinement EMD-51400	Conformer 2 ORC local refinement EMD-51399	OC_1M_{Mcm5RA} conformer 2 global refinement EMD-51401 (composite) EMD-51398 (consensus) PDB 9GJP
**Data collection and processing**				
Magnification	130,000	130,000	130,000	130,000
Voltage (kV)	300	300	300	300
Electron exposure (e^-/Å^2)	30.34	30.34	30.34	30.34
Defocus range (μm)	-1.5 to -2.7	-1.5 to -2.7	-1.5 to -2.7	-1.5 to -2.7
Pixel size (Å)	0.95	0.95	0.95	0.95
Final particle images (no.)	49,788	103,314	151,030	151,030
Map resolution (Å) at 0.143 FSC threshold	3.67	3.47	3.27	3.39
**Refinement**				
Model-map CC[a,b] (CC_{mask}/CC_{box}/CC_{peaks}/CC_{volume})	0.69/0.80/0.57/0.68			0.71/0.80/0.60/0.70
Model resolution (Å) at 0.143 FSC threshold[a,b] (masked/unmasked)	3.7/3.7			3.4/3.5
Model resolution range (Å)[c]	3.2-14.0			3.0-14.0
Model composition				
Non-hydrogen atoms	52,641	50,292		
Protein residues	6,331	6,036		
Ligands	2 ATP, 4 ADP, 5 Zn^{2+}	2 ATP, 4 ADP, 5 Zn^{2+}		
B factors (Å^2)[a]				
Protein	247.70	244.57		
Ligand	152.56	137.92		
R.m.s. deviations[a]				
Bond lengths (Å)	0.002	0.002		
Bond angles (°)	0.546	0.547		
Validation[d]				
MolProbity score	1.18	1.32		
Clashscore	3.66	4.52		
Poor rotamers (%)	0.02	0.00		
CaBLAM outliers (%)	0.92	1.16		
Ramachandran plot[d]				
Favored (%)	97.89	97.54		
Allowed (%)	2.11	2.46		
Disallowed (%)	0	0		

[a] Comprehensive validation (cryo-EM) in Phenix[6]. [b] Values shown are for the consensus map, which was used for the refinement. [c] Estimated with Chimera 'Values at atom positions' tool[7].

[d] MolProbity[8] validation in Phenix.

Supplementary References

1. Punjani, A., Rubinstein, J.L., Fleet, D.J. & Brubaker, M.A. cryoSPARC: algorithms for rapid unsupervised cryo-EM structure determination. Nat Methods 14, 290-296 (2017).
2. He, J., Li, T. & Huang, S.Y. Improvement of cryo-EM maps by simultaneous local and nonlocal deep learning. *Nat Commun* **14**, 3217 (2023).
3. Yuan, Z. et al. Structural basis of Mcm2-7 replicative helicase loading by ORC-Cdc6 and Cdt1. Nat Struct Mol Biol 24, 316-324 (2017).
4. Sagendorf, J.M., Berman, H.M. & Rohs, R. DNAproDB: an interactive tool for structural analysis of DNA-protein complexes. Nucleic Acids Res 45, W89-W97 (2017).
5. Lim, C.T. et al. Cell Cycle Regulation has Shaped Budding Yeast Replication Origin Structure and Function. bioRxiv (2024).
6. Liebschner, D. et al. Macromolecular structure determination using X-rays, neutrons and electrons: recent developments in Phenix. Acta Crystallogr D Struct Biol 75, 861-877 (2019).
7. Pettersen, E.F. et al. UCSF Chimera - a visualization system for exploratory research and analysis. J Comput Chem 25, 1605-12 (2004).
8. Chen, V.B. et al. MolProbity: all-atom structure validation for macromolecular crystallography. Acta Crystallogr D Biol Crystallogr 66, 12-21 (2010).
