# Supplementary material for: Unidirectional MCM translocation away from ORC drives origin licensing
Source: Nat Commun. 2025 Jan 17;16:782. doi: 10.1038/s41467-025-56143-y (PMC11748629; doi:10.1038/s41467-025-56143-y)
Supplement: Supplementary file 2 — Description of Additional Supplementary Files [file 41467_2025_56143_MOESM2_ESM.pdf]

Description of Additional Supplementary Files

File Name: Supplementary Movie 1
Description: Comparison of ATPγS-OCCM, $OCCM^{Mcm2RA}$ and OC_1M^{Mcm5RA} structures. Morphing reveals the sequential DNA engagement of neighbouring Mcm2 and Mcm5 subunits away from ORC/Cdc6.
